# Editorial

**Published:** 1851-06

**Authors:** 


### THE MEDICAL EXAMINER.

PHILADELPHIA, JUNE, 1851.

[The facts presented in the subjoined statement, have been submitted to us, with a request that we would embody them in an editorial notice, with the assurance that they have the approval of both parties. It affords us much pleasure to comply with this request, and to learn that this correction of unintentional mis-statements terminates a regretted controversy, between gentlemen whose previous relations had been uninterruptedly cordial.—EDS. EXAMINER.]

### EXPLANATIONS AND CORRECTIONS.

Dr. Jackson, in his "Review of the Memorial of Dr. John Bell to the Trustees of the University of Pennsylvania," writing in haste, and under what he felt to be no small provocation, was betrayed into some errors of fact, which it is desirable to correct. These corrections are due, in justice, to Dr. Bell, while, at the same time, they do not touch Dr. Jackson's reputation on any point in previous controversy. They are now made with the knowledge and assent of both these gentlemen.

Dr. Jackson (in his "Review") states that "Dr. Bell was a lecturer for about ten years in the Philadelphia Medical Institute." Dr. Bell was, in fact, a lecturer in that institution during an uninterrupted period of *nineteen* years, in addition to *two* years as a member of the Association, before it was organized under the title of the Medical Institute.

Again, it is stated, in the "Review," "He [Dr. Bell] was for some six or eight years a lecturer on the same subject, [the Institutes of Medicine."] He was, actually, a lecturer on this subject *seventeen* years, not to speak of *two* years antecedently, in which he lectured on Physiology and Hygiene. During sixteen years of this period, he gave, also, lectures on Medical Jurisprudence, in connection with those on the Institutes of Medicine.

Dr. Jackson's memory is at fault, when he alleges that—"Alterations were made in the management of the association [the Medical Institute], in order to favor Dr. Bell and to place him, at a sacrifice to his associates, on a pecuniary footing with themselves." Doctor Jackson's subsequent explanation of this averment is, that "In the first years of the courses of the Institute, the lecturers had the privilege of sending their private students free of charge to the Institute," but that, subsequently, the rule was altered, so as to require the fees of these students to be "paid into the treasury of the Institute and a dividend made equally." Dr. Jackson's recollection of the change, as here represented, is, that it was to favor Dr. Bell, the number of whose students was, Dr. J. thinks, small, "while the other lecturers had numerous pupils."

The change actually made, as we learn by reference to the records of the Institute, was, in requiring each lecturer, who had a private, or as the term, on the records, is, "perpetual" student, to pay into the treasury of the Institute, one hundred dollars, by which he, the student, acquired the privilege of attending all the lectures and examinations in the Institution, during the entire period of his medical studies up to the date of his graduation. Each lecturer was allowed to receive, as before, the fees for three annual students, who entered with him for the year, or who were alloted to him, if they did not make personal application. The other students of the Institute were they who enrolled themselves at the beginnning of each scholastic year, and took a ticket, for this period, for the full course, that is for attendance on all the lecturers. The money received for this ticket had always been deposited in the treasury for equal division among the lecturers. Whatever may have been the motive for the change in the rule of the Institute, respecting private pupils, it is very certain that the one alleged by Dr. Jackson, could not have been, at all, a determining one. This will be apparent when we state that Dr. Bell was less in a position, at the time, to require any special legislation, in this matter, than most of his associates. The new rule was adopted in March, 1830. Now, the records of the Institute show, that Dr. Bell, in the year preceding, or 1829, had more private pupils, in addition to his three annual ones, than any other member of the Institute except Drs. Chapman and Mitchell. His private pupils, in that year, numbered *four*; those of Dr. Horner, three; of Dr. Dewees, two; of Dr. Jackson, two, and of Dr. Thomas Harris, one. Dr. Hodge does not appear to have had any. In the year 1828, Dr. Bell had *five* private pupils—Dr. Dewees, none.

There were great fluctuations in the number of private pupils, from year to year, in the offices of the different lecturers. It was thought that there might be more equality established for all, in this matter, and hence the new rule, which continued in operation to the time when the Institute was dissolved; with the modification, in later years, of requiring only seventy dollars from a lecturer for his private pupil; no engagement being made for winter examinations as before.

No favor was ever asked by Dr. Bell from his associates in the Institute, nor was any ever conceded to him, by these gentlemen, in the way of lightening his labors. The change in his position as a lecturer in the Medical Institute, three years before the association was dissolved, may be adverted to on the the present occasion. It was made at the request of Dr. Jackson and for his convenience. It consisted in Dr. Jackson's taking the *Institutes of Medicine*, and Dr. Bell *Materia Medica and Therapeutics*, so as, thereby, to allow of the former of these gentlemen to continue without interruption, during the summer session of the Institute, the lectures and their associated reading and study, in which he was engaged during the winter, in the University.

Dr. Bell acceded to Dr. Jackson's request, although, from its having been made only about a month before the opening of the lecture session of the Medical Institute, he would be placed at a great disadvantage in beginning a course of lectures on a new subject, without time for preparation. It so turned out, however, that Dr. Bell succeeded in obtaining the approbation of the students and of other occasional auditors, who attended his lectures on Materia Medica and Therapeutics—during the three years which preceded the dissolution of the Institute, as it was then organized. The broad assertion in the "Review," that, "During the whole period, Dr. Bell was attended by a mere fraction of the class," must be met by direct denial. If ever applicable, it could only have been so during the time when Dr. Gerhard gave clinical lectures in the Blockley Hospital. Dr. G.'s hour was that of Dr. Bell's lecture, and the students very naturally preferred clinical to didactic instruction.

On this point, it is but justice to Dr. Bell to say, that, in the year 1837, he received an offer of the Chair of the Practice of Medicine in the University of Transylvania. The offer was made to him by a member of the Faculty of that school, immediately after the latter had heard Dr. Bell give a lecture, (one of his regular series,) in the Medical Institute. The Transylvania professor was, himself, of the highly rhetorical school of lecturing, and his manner, in this respect, must have been in strong contrast with the plain style of composition and delivery of Dr. Bell.

At a subsequent period, Dr. Bell received an offer, on the part of the Faculty of Franklin Medical College, of Philadelphia, of the Chair of the Practice of Medicine in that Institution, which had been left vacant by the resignation of Dr. Clymer. The fact is noted here, because two of the professors of the college had been associates of Dr. Bell in the Medical Institute.

Dr. Jackson, after making the general assertion, already disposed of, that Dr. Bell had been specially favored by his associates in the Institute, at a pecuniary sacrifice to them, adds: "At last those who had the most work to do found the compensation so trifling, that the organization of the institution was dissolved." The fact of dissolution and its chief cause are correctly stated; but if we were to ask,—who had the most to do? the reply must necessarily be, Dr. Bell, inasmuch as he was the only member of the Institute, at the time, who had not an assistant to lighten his labors. In the last session of the Institute, there were no less than thirteen gentlemen engaged in teaching its several branches—seven in number. Even were we to suppose a deficiency on the part of any one of the original seven lecturers, this would be compensated by the numerous assistants or adjuncts, some of them of marked ability, all of them fully competent to perform their share of the duties assigned to them.

The gradual decline of the Institute classes may have been produced by various causes. The most obvious one was the diminished interest which the Professors of the University, who constituted a majority of the associated lecturers, took in the Institute; as evinced by the fact of their failing to give the same time and attention to it, which they had given in its more flourishing days. We cannot be accused of detracting from the merits and zeal of those gentlemen who were associated with the Professors in the business of summer teaching, when we express our belief, that the great attraction for the students who enrolled themselves in the Institute classes, was the privilege of hearing these latter repeat and enlarge on their winter lectures. The students believed that by this means they would, as, in fact, they did, obtain increased facilities for reaching the doctorate with credit. In making this remark, we would not have it implied that we advocate a participation in summer teaching by the Faculty, or members of the Faculty, of any regularly incorporated Medical College, in which a winter session is held.

On another topic Dr. Jackson has allowed himself to make a grave charge against Dr. Bell. When speaking of the latter as "ambitious of the reputation of authorship," Dr. Jackson says: "But when the principal alteration and addition consists in the erasure of the author's name on the title page, and the substitution of his own, some other appellation might be given to the act." Dr. Jackson could not have seen the work to which we supposed, and now know, he refers; and he must, we are sure, deeply regret that he has been betrayed into so serious an accusation against another person, without his having assured himself that there were, at least, some grounds for making it.

There is only one work under Dr. Bell's name, the title of which makes a near approach to that of another author. It is the *Practical Dictionary of Materia Medica.* Mr. Brande's work bears the title of *Dictionary of Materia Medica and Practical Pharmacy.*

The title page of Dr. Bell's volume exhibits, quite conspicuously, an acknowledgment of its being on the basis of Mr. Brande's. Hence, at the very first glance, the reader is apprised of the source from which the American author might be supposed to derive his chief materials. But, a still more precise and comprehensive explanation, on this point, is given by Dr. Bell in the Preface, in which, also, he assigns the reasons why Mr. Brande's name could not be retained on the title page, without positive injustice to this gentleman. Dr. Bell made, not only many curtailments of the English work, but, also, many additions, and, still more, organic changes in it, so that its chief features were greatly altered.

The additions made by Dr. Bell, as stated in his Preface, "are interwoven, in nearly every article, with the summaries of Mr. Brande; and often, without their taking up much additional space, they give a different complexion to the opinions which he had advanced, but one which is more in consonance with clinical experience. Some articles which had a place in the English work have been replaced, on account of their meagreness and incompleteness, by other more extended ones, prepared by myself. Of these I may mention *Emetics*, *Emmenagogues*, *Epispastics*, *Hirudo*, and *Iodinum*, including under this latter the combinations of iodine with *carbon*, *sulphur*, *arsenic*, and *mercury* and *arsenic*, none of which were noticed by Mr. Brande. To the articles under the heads of *Antimony*, *Ferrum*, *Plumbum* and *Zincum*, the additions have been considerable, either in their novelty or amount, as compared with the original copy. Quite a different view of the therapeutical powers of antimony is given from that advanced by Mr. Brande. Neither the *lactate of iron*, nor the *carbonate*, nor *iodide of zinc*, was mentioned by him; and the remarks on the disease caused by the poisoning of lead, belong to the American work." Among the additions introduced by Dr. Bell, are twenty-eight articles of the indigenous Materia Medica of the United States.

While specifying the retrenchments which he made, such as of chemical details, diagrams, and of botanical descriptions of plants and their ultimate analysis, Dr. Bell takes care to point out to the reader in what respects his work is on the basis of Mr. Brande's, and to indicate the subjects retained, including the extracts from Dr. Paris's *Pharmacologia*, and Dr. A. T. Thomson's *London Dispensatory*, which had been freely introduced into the English volume.

The Dictionary was brought out by Dr. Bell with less preparation that would otherwise have been given to it, owing to his having announced Mr. Brande's volume for publication in his *Select Medical Library*, on the strength of the interest of the subject, and the reputation of the author. When, however, he had an opportunity of perusing Mr. B.'s work, he soon saw that it would not answer; but as there was no other volume at hand to meet the requirements of the case, viz., republication at an early given date, he set about making what he deemed either necessary or desirable changes, and, in a measure, reconstruction, within the very short period allowed him for the purpose.

Were Dr. Bell not desirous of preventing farther controversy, he might retort, with some force and point, on Dr. Jackson, for the harsh strictures in which the latter has indulged in his "Review." But he forbears, under a belief, we might add, an assurance, that the feelings which prompted these strictures are no longer entertained; and he himself has no wish to yield to similar ones. With this understanding, and the explanations and corrections now made, Drs. Bell and Jackson are willing to let things take their old course.

*May 19th*, 1851.

---

DEATH OF SAMUEL GEORGE MORTON, M. D.

It is with the greatest regret that we record the death of Samuel George Morton, M. D., which took place in this city, on the 15th of May last. Although in feeble health for many years past, and evidently holding life by a very slight tenure, Dr. Morton was in the discharge of his ordinary professional duties, up to the period of his last illness, which was of only four days' duration. His final attack was of an apoplectic nature; but he had long labored under considerable disease of the lungs and heart, as will be seen from the autopsy, appended to our notice, for the details of which we are indebted to Dr. Neill.

The death of Dr. Morton deprives our profession of one of its most distinguished members, and abruptly terminates a scientific career, which had become one of the brightest illustrations of our country. His reputation as a naturalist was diffused throughout the world, and we believe that no cotemporaneous American name was better known abroad than his. His loss, in every respect to be lamented, will be particularly felt in the department of natural history, in which he was prosecuting the most interesting researches, when thus prematurely cut off, at the age of fifty-two, in the prime of mental vigor and usefulness. In the relations of private life, no one ever conciliated more universal esteem and affection than Dr. Morton. He passed through the world, literally without an enemy, beloved and respected in all circles, everywhere deservedly regarded as one of the most amiable and irreproachable of men.

Dr. Morton was a native of Philadelphia, but, we learn, passed a portion of his youth in New York. He pursued his medical studies in the University of Pennsylvania, and in the office of the late Dr. Parrish. After graduating in the University of Pennsylvania, he spent several years in Europe, and received a second degree from the University of Edinburgh.

Returning to his native city, Dr. Morton rapidly passed into extensive practice, and, up to the time of his death, enjoyed a popularity second to none among the practitioners of Philadelphia. To the literature of his profession he made numerous valuable contributions. In 1835 he edited Mackintosh's Practice of Physic, which went through three subsequent editions; in 1833 he published an excellent original work on Consumption; and in 1849 an elaborate work on Anatomy.

Dr. Morton was for many years engaged as a teacher of medicine, and always ranked among our best lecturers. He gave several clinical courses in the Philadelphia Almshouse Hospital, and was Professor of Anatomy in the original organization of the Pennsylvania Medical College.

With this assiduous and unfaltering devotion to strictly professional avocations, Dr. Morton combined an enthusiastic and earnest pursuit of natural history, which earned for him a European reputation, and gave many splendid results to science. His first scientific publication was a work on the *Fossils of the Cretaceous Group*, in which, we believe, he described every example that has been found in N. America.

As far back as twenty years ago, he commenced collecting the materials, which he eventually embodied in the *Crania Americana*, the first work of the kind ever produced. This great work, published in 1839, immediately placed the author in the foremost rank among the cultivators of natural science, and was received throughout the world as a most valuable original contribution to ethnology. Its full importance is yet to be appreciated. At present it stands almost isolated; and its real value can be developed only when other races of men are studied and tabulated on a similar plan, and a comparison of the averages afforded, enables us to determine the characteristic differences between the various races.

Dr. Morton spent many years upon the Crania Americana, and so careful was he to produce it free from inaccuracy, that, after the elaborate tables had been nearly completed—upon Mr. George Combe's pointing out a slight error in the starting point from which the measurements had been made—a new series was at once commenced, and carried through on the corrected method.

The *Crania Egyptiaca* followed the *Americana.* The presence of the integuments, however, and the bituminized condition of the crania, prevented any definite series of measurements.

The subject of hybridity occupied much of Dr. Morton's attention in the latter period of his life. At the time of his death he was pursuing his inquiries in several interesting and hitherto unexplored channels, connected with this important branch of natural history; and he had already collected a vast number of facts, and reached the solution of many obscure and previously unnoticed points. In this course of investigation, he was led into a close examination of the specific characters of the wolves of N. America, and the results of the crosses between the different species of wolves and the imported dogs,—a thread of inquiry which we know was developing most valuable conclusions in the highest walks of natural science.

In the midst of these absorbing scientific and professional labors, Dr. Morton found time to indulge a taste for poetry; and his occasional effusions show that he united a fine imagination, and refined appreciation of the beautiful, with his more solid powers and attainments. And all these noble intellectual qualities were graced with the crowning attractions of a most unaffected bearing, the gentlest manners, and a genuine cordiality and kindliness of disposition!

At the time of his death, Dr. Morton was President of the Academy of Natural Sciences, of which he had been a leading member for thirty years. He was also a Fellow of the College of Physicians, of the American Philosophical Society, and of numerous other learned societies, at home and abroad. The various associations with which he was connected in Philadelphia, united in every possible tribute of respect to his memory.

*Post Mortem Examination of Dr. Samuel G. Morton.*

On the day previous to his death, Dr. Morton was considered convalescent from an indisposition which his physicians had not supposed to be dangerous or alarming.

About the middle of the same day, a tendency to stupor was noticed, which gradually increased, and terminated in paralysis and death.

Present—Drs. Rodman, Beesley, Wistar, Pepper, McClellan and Neill.

*Head.*—The symptoms immediately preceding death, directed attention particularly to the condition of the brain, it being supposed that effusion had taken place in that organ. The arachnoid had lost its transparency, and no fluid was found in its cavity, but there was considerable serous effusion in the sub-arachnoid cellular tissue.

The pia-mater was very much congested, particularly on the left side, and from its vessels blood had been extravasated in several places. The basilar and vertebral arteries contained venous blood. Two small clots were found very nearly in similar positions upon the superior surface of each hemisphere ; the anterior clot was the larger upon either side, and rested upon the upper surface of the anterior lobe. It consisted of about one drachm of blood, and had assumed the form of the sulci, into which it had insinuated itself. The brain itself was large and symmetrical. Its substance was firm and natural in every respect. The choroid plexus was equally congested with the pia-mater. Very little fluid was found in the ventricles.

*Thorax.*—The pericardium contained the usual amount of serum, and presented no appearance of disease.

The heart was large and flabby, and its tissue was pallid and somewhat softened. The cavities generally were dilated; the parietes of those of the right side were particularly thin, and contained some fibrinous clots. The ostium venosum of the right side was enlarged to such a degree that it could not have been closed by the tricuspid valve. The mitral valve was much thickened by soft fibrinous yellow deposit.

The arch of the aorta was dilated, and had some small patches of atheromatous matter deposited upon its internal surface.

The left lung was entirely useless for the purposes of respiration. It was contracted to a very great degree, and occupied but a small space in the upper portion of the thorax, where it was firmly bound down by the adhesion of the costal and pulmonary pleura, by which the cavity of the pleura was entirely destroyed. The congestion of the lung was so great, that it appeared dark-colored and solidified, although it contained sufficient air to make it float in water.

The right lung, which was unusually large, was congested and adherent to the walls of the thorax by adventitious bands of an old formation. The cavity of the pleura contained about a half pint of bloody fluid, probably the result of post-mortem changes.

These adhesions of the left pleura, and the contraction of the left lung were produced by an inflammatory affection of these organs, which occurred a few years since.

*Abdomen.*—The spleen was enlarged and very much congested. The tissue was so soft and pulpy that it readily yielded to pressure by the finger.

The liver was natural. The alimentary canal was not examined.

It can readily be understood from the above facts, that the immediate cause of Dr. Morton's death was *meningeal apoplexy*, resulting from that disturbance of the circulation which was manifested by the engorgement of the blood-vessels of the brain and lungs, but which was produced by dilatation of the heart.

---

### AMERICAN MEDICAL ASSOCIATION, PRIZE ESSAY.

The committee, appointed under the resolution appended to the report on medical literature for 1849–50, have awarded the prize of *one hundred dollars, or a gold medal of equal value*, to Dr. J. C. Dalton, of Boston, for his essay "*On the Corpus Luteum of Menstruation and Pregnancy.*" Francis G. Smith, M. D., *Chairman.*

---

Professor Mütter sailed for Europe on the 24th of May, with the intention of spending the summer months abroad. We hope to receive from him an occasional letter for our columns.

